# The concerning rise in hypertension among children and adolescents

**DOI:** 10.1016/j.eclinm.2026.103777

**Published:** 2026-01-23

**Authors:** eClinicalMedicine

Typically recognised as a disease affecting middle-aged and older adults, hypertension rates among children and adolescents are on the rise. According to a recent systematic review and meta-analysis of studies involving over 440,000 children from 21 countries worldwide published in *The Lancet Child and Adolescent Health*, the global prevalence of hypertension among children and adolescents aged 19 years or younger has nearly doubled among boys and girls between 2000 and 2020. The study estimated a pooled prevalence of childhood hypertension of 4.28% (95% CI 3.71–4.90) based on in-office measurements, and a prevalence of sustained hypertension of 6.67% (1.66–14.53) based on the combined in-office and out-of-office approach. For sustained hypertension, higher prevalence rates were observed in low-income and middle-income countries (LMICs; 8.13% [95% CI 4.75–12.30]) compared with high-income countries (3.70 [1.85–6.12), and the highest estimates were observed in the WHO Americas region (11.67% [95% CI 4.02–22.53]), indicating regional disparities. However, heterogeneity between studies (in terms of study design, population characteristics, blood pressure measurement protocols, and applied hypertension guidelines), and imbalanced regional coverage were noted in the study, indicating a lack of standardised measurement strategies, diagnostic gaps, and unclear estimates in certain countries or regions. These findings signal the concerning current and growing global burden of childhood hypertension, gaps in our understanding of the extent of the problem, and the need for urgent action to protect the health of children.

Hypertension is a major cause of premature death worldwide, and an elevated blood pressure in childhood is significantly associated with hypertension in adulthood. In children, hypertension can be caused by renal, vascular, neurological, or oncological diseases, endocrine disorders, genetic factors, as well as medications and toxins, but other risk factors might also be contributing to the growing burden of the disease. In the aforementioned global meta-analysis, childhood hypertension prevalence estimates varied significantly across BMI groups, with the highest estimates found in children and adolescents with obesity and overweight. In children, the risk of hypertension is estimated to be around 2.6 times greater among those with overweight, and around 9.2 times greater among those with obesity. Similar to the increasing trends of childhood hypertension, the combined global prevalence of overweight and obesity among children and adolescents has doubled between 1990 and 2021, with 15.6% of children aged 5–14 years predicted to have obesity by 2050. Dietary habits and behavioural factors could also have a role in hypertension risk. Around 80% of adolescents do not meet the recommended levels of physical activity, with sedentary behaviour and prolonged screen watching associated with high blood pressure among children and adolescents. As with adult hypertension, high salt intake is also positively associated with increased blood pressure in children and adolescents, with an estimated 73% of children exceeding recommended sodium intake levels globally. Around three-quarters of salt consumed comes from packaged products, such as processed meat, bread, and savoury sauces. Although there is limited evidence, exposures such as stress, smoking, air pollution, and social determinants of health are also thought to present modifiable risk factors for childhood hypertension.

Childhood hypertension can have short-term and long-term adverse effects on kidney, heart, eye, and brain health. Compared with normotensive children, those with primary ambulatory hypertension show increased odds of target organ damage, including left ventricular hypertrophy, and elevated pulse wave velocity and carotid-intima-media thickness. Elevated blood pressure during childhood and adolescence has also been associated with an increased risk of these cardiovascular outcomes or stroke during adulthood. In children with arterial hypertension, increased carotid intima-media thickness is associated with changes in optical coherence tomography angiography parameters, indicating that remodelling of the retinal microcirculation can also occur. Regarding neurological effects, hypertension in children and adolescents could be associated with decreased performance on neurocognitive tests and deficiencies in executive functioning in early life. Additionally, a retrospective, population-based cohort study in Canada showed that the risk of major adverse kidney events (defined as all-cause mortality, incident chronic kidney disease, or kidney failure) was around three times higher among children and adolescents with hypertension compared with matched controls without hypertension during a median of around 14 years’ follow-up.

Addressing the issue of rising childhood hypertension and protecting children from the detrimental effects on their health requires a multi-level approach. Routine monitoring of blood pressure in children is necessary to understand the true burden of childhood hypertension and raise awareness of the condition. Guidelines recommend screening for hypertension of children at preventive care visits, and more frequent screening among children and adolescents at increased risk of cardiovascular disease. However, evidence suggests that screening rates for children and adolescents are insufficient and compliance to recommended blood pressure measurement practices is poor, increasing the risk of misdiagnosis. Furthermore, there is a lack of hypertension screening policies in LMICs, and differences in blood pressure measurement protocols, hypertension thresholds, and blood pressure targets across existing screening guidelines. A universal screening programme, standardised blood pressure measurement protocols, and harmonised diagnostic criteria are needed to identify children and adolescents with hypertension and enable early intervention. In a positive step forward, UK doctors recently called for a national programme to monitor schoolchildren for high blood pressure due to concerns about the long-term adverse health effects of rising childhood hypertension rates.

Supporting efforts to avoid the consumption of processed foods high in salt, saturated fat, or sugar, and increase physical activity (particularly aerobic activity) and reduce screen time among children and adolescents could also play an important role in preventing and managing childhood hypertension. Adherence to a Dietary Approaches to Stop Hypertension-style eating pattern has been associated with a lower systolic blood pressure throughout adolescence. Evidence also suggests that interventions that combine physical activity with nutrition and behaviour modification components might be most effective at reducing both systolic and diastolic blood pressure in children and adolescents. Engagement of both caregivers and health-care providers is important for implementing such interventions, but barriers such as cost, education, and availability and access to healthy foods should also be considered. At community and population levels, providing school meals that are nutritionally balanced and contain minimal and no added salt, sugar, or saturated fat, and restricting advertising of less healthy food and drinks to children on television and online could also help to reduce the consumption of excess calories and salt in this age group. Context-dependent community and clinical interventions to tackle the social determinants of health related to hypertension are also needed to address disparities in blood pressure control and create environments that encourage healthy behaviours across the life-course.

The growing burden of childhood hypertension is a cause for global concern that, if left unaddressed, could have substantial and potentially irreversible effects on children's health. Implementation of blood-pressure screening and surveillance for children worldwide is needed to fill data gaps and ensure elevated blood pressure does not go undetected or untreated. By combining lifestyle and behavioural interventions with public health policy measures, governments, schools, health systems, and communities can help to curb the rising childhood hypertension rates and protect children from future cardiovascular and renal disease.

